# Transforming professional identity of medical teachers in Pakistan by a certificate program in health professions education: a thematic analysis of reflective essays

**DOI:** 10.3389/fmed.2023.1323075

**Published:** 2024-02-21

**Authors:** Faiza Kiran, Rukhsana Ayub, Ayesha Rauf, Asiya Zahoor

**Affiliations:** ^1^Army Medical College, National University of Medical Sciences, Rawalpindi, Pakistan; ^2^NUMS Department of Health Professions Education, National University of Medical Sciences, Rawalpindi, Pakistan

**Keywords:** professional identity, medical teachers, faculty development, teacher education, professional development

## Abstract

**Introduction:**

A teacher’s professional identity development is a dynamic and continuous process that requires rigorous, longitudinal, faculty development initiatives which are designed to work on individual professional growth. Impact of such programs must be evaluated by qualitative means to identify the process of change; The purpose of our study was to investigate whether and how our thoughtfully designed 6-month certificate program has transformed the professional identity of medical teachers.

**Methods:**

The study was conducted in National University of Medical Sciences, Pakistan using thematic analysis, wherein a weekly task of guided reflective writing, on Moodle, was given. Data was analyzed in six phases to achieve credibility and transferability.

**Results:**

By analyzing 202 reflective writings, seven subthemes were identified which manifest transformation in certain aspects of identity of participants and grouped under three major themes. The seven subthemes represent transformative journey of participants and include recognizing millennial learners’ dilemma, identifying learning gaps and overcoming barriers, discovering a newer version of self, alternative frame of thinking, transforming traditional classroom, conducive learning environment and Community of Practice. Three major themes identified were Awareness, Modeling and Socialization which represent three processes bringing transformation in participants.

**Conclusion:**

Our faculty development program has transformed certain aspects of professional identities of medical teachers by incorporating informal teaching strategies of experiential learning, professional socialization, reflections, and role modeling. Participants’ beliefs and practices on teaching were challenged by giving a disorienting dilemma of millennial learners and learning theories. They underwent critical discourse with professional peers and mentors in community of practice, reflected on their traditional teaching practices, acquired new insight, underwent self-discovery, and introduced digitalization and interactive learning strategies within their classrooms.

## Introduction

Health Care Professionals (HCP) often perform multiple roles as clinicians, researchers, and educators. Their identity as a clinician and a scholar is supported and developed early in their professional careers but they often struggle to develop their identity as a teacher (1). A teacher as a professional must be proficient in the subjects’ content, instruction, and assessment to achieve curricular goals (2). Faculty development programs (FDP) can play a strategic role in fostering and strengthening a teacher’s identity in addition to skills (1). Thus, institutions invest hugely in professional development endeavors. Unfortunately, the format of sustained, intensive, job-embedded professional development in the past, neither brought improvement in knowledge of teachers nor in instruction or students’ achievements (3).

Identity encompasses how individuals understand themselves, interpret experiences, present themselves, wish to be perceived by others, how they interact and are recognized by the broader community (4). A teacher’s professional identity development is a dynamic, ongoing, and a socially situated, constructivist phenomenon, continuously shaped and reshaped in a constant dialogue with people within their social environment (5, 6). This identity is depicted in ways they narrate their experiences (3). Professional identity formation involves acquiring core professional values, set of behaviors, and moral principles of members of community (7). This is achieved by taking inspirations from role models, mentoring, feedback, and charting own progress towards membership of community by ongoing self-reflections (8).

Literature states that formation of strong professional identity of teachers enhances their intention to stay in their profession, increases their willingness to invest their time, energy, and resources in their professional development, and facilitates them in their teaching role (5). The well-being of medical educators is related to their strong identity development, thus reducing the risk of burn out by promoting resilience (7). Crucial to identity development is the role of community of practice where teachers interact with their peers, experience relatedness, and learn through each other’s experiences via process of socialization (8). This concept has been first proposed in 1991 by Lave and Wenger who emphasized role of community of practice (CoP) on identity construction of a *novice* into an expert by mutual sharing of ideas and experiences with senior members of community, eventually leading towards their transformed identities (9).

Though different forms of participation and interaction within a community supports teachers’ identity development, it is argued that engagement, alignment, and imagination are three important distinct modes of participation. Engagement is the result of a teacher’s direct pedagogical function, for example, working with senior colleagues and administrative staff, attending institutional meetings, or following educational polices. Teachers not only participate within the boundaries of physically located communities, but they also belong to broader professional networks. This is defined as alignment. Another form of participation is represented as imagination, which accounts for a teacher’s sense of individuality which is reflected in forms of innovation and creativity (9, 10). There arises an idea to develop a program which creates opportunities to create all three types of participations within members of community, where professionals meet, interact, reflect, create self-awareness by transforming abstract and theoretical knowledge into something meaningful, personal, and unique (8–11). The process is augmented when an enabling environment is fostered, and feedback and mentorship is inherently practiced (11).

Those faculty development initiatives which employ peer interactions and group activities within CoP, are structured and of longer duration as compared to fragmented one-time events, transform professional identities of teachers (12). However, a recent review study by Kohan et al. states that the majority worldwide faculty development programs for health professionals in past 30 years were workshops (33%), seminars (13%), and short courses (16%) and of these, mentorship was present in only 5% of programs (13). Literature is also deficient where the faculty development activities focus on professional identity development of faculty members. Most programs neither incorporate informal learning tools of role modeling, experiential learning, peer interactions nor formal personal development activities necessary for identity development such as reflections, feedback, and online learning. The design and delivery of these faculty development programs is committed to enhance the traditional role of faculty members as only the transmitter of information. However, promoting teaching effectiveness, educational leadership, and organizational change, capitalizes largely on systematic planning of fostering professional identities of teachers, by introducing experiential learning, reflecting on that experience, workplace learning and learning within community of practice. This provides opportunities to build strong community networks and maximize modeling and mentorship (13).

Literature of our country tells us that our most faculty development programs are literary, didactic, non-evidence-based with no physical interaction, launched without assessing the needs of the faculty, hence, they just reiterate the old teaching practices at workplace and fail to transform the traditional mindset of educators (14). Moreover, many clinical teachers believe that being a good physician means that they are good teachers, too (15). Of special concern is the recent boom in the 2 years’ Master program and Certificate courses of six-month duration in Health Professions Education (HPE), for which none of the National regulatory bodies provide the minimum standards (14), raising serious concerns on their quality and that of graduating faculty members. One cross sectional study on online Certificate in Health Professions Education (CHPE) involving eight participants reported overall satisfaction with the program but identified internet issues, poor teaching skills in a virtual environment and lack of face-to-face session as main challenges (16). Research on medical teachers’ identity in Pakistan is also scarce. The recognition of these scarcities compelled us to design a properly structured, evidence based, longitudinal faculty development program, focused on development of professional identity of our medical teachers. The program was based on foundational theory of Community of Practice which implies learning as a social activity, situated within community dynamics (8, 9). However, educational activities, that are designed and employed within this community of practice, are adopted from other learning theories; an approach referred to as an integrated theoretical approach (8). This concept has also been endorsed by Kaufman and Mann who in their review of learning theories in medicine, stated that an advantage of situated learning theory, which is derived from social learning theory, is its ability to relate to and incorporate other learning theories (17).

Community of Practice has a powerful impact on learning and identity formation of its members. The strength of the identity formation process is its dynamic nature where identity is socially constructed and remodeled via an ongoing process of negotiations among new and older members of community, a process called legitimate peripheral participation (8, 9). Wald has described this process of identity formation as a transformative journey where personal and professional growth of a professional is fostered through informal teaching activities such as mentorship, self-reflection and experiential learning (7, 13). The goal of our program was to transform the identities of our participants by giving them a platform of interprofessional community of practice, where their preconceived notions of traditional teaching practices are challenged by mentors and role models, they acquire tacit knowledge by interacting with facilitators and peers in specifically designed tasks, share their beliefs, reflect, and transform their identitis (18).

Global literature is replete with outcome-oriented approach in studies evaluating impact of faculty development programs and using quantitative data collecting tools such as surveys, pre- and post-tests. However, to understand how individual and organizational change occurs because of faculty development intervention, process-oriented approaches are needed (12). Therefore, when we aimed to evaluate our University CHPE program by appraising its impact on the professional identity of participants, we decided to take a process-oriented approach. The research questions were “Whether” and “How”, our CHPE program has transformed the professional identity of medical teachers?

## Materials and methods

### Context

In 2019, we developed and launched a six-month Certificate in Health Professions Education (CHPE) at National University of Medical Sciences, Pakistan. With nine medical, three dental and three nursing colleges and one post graduate institution, the total number of faculty members of university is over 3,000. Since the courses were to be disseminated in other institutions, a scalable, sustainable faculty development program needed to be developed. Thus, a hybrid program was designed to translate theory into practice with a major focus on interprofessional socialization, supportive environment, and experiential learning (8, 9). The goal was to challenge old held beliefs of traditional faculty, develop their personal insight and self-awareness, by evidence based transformative tools of learning such as modeling, mentoring, feedback (7, 8, 13, 18).

A total of four sequential courses were developed: Adult Learning Theory and Application (ALTA), Curriculum Planning, and Evaluation (CPE), Evidence Based Teaching and Learning (EBTL) and Assessment for Learning (AFL). The first two courses last for 5 and the last two courses last for 6 weeks. The courses were designed utilizing the principles of adult learning (19, 20). Adult learning theories were recognized as a foundation for learning health professions education through literature search (19, 20) and extensive discussions among our team members. Consequently, the first course developed by the first and second authors was Adult Learning Theories and Application (ALTA), which was run on Moodle; an online learning management system used for distant learning. Four major categories of learning theories (19) were introduced during this course; Instrumental (Behaviorism, Cognitivism), Social (community of practice, situated learning, sociocultural), Motivational (Self-Determination Theory; Attribution Theory; Maslow’s Hierarchy; Transformational learning; Self-Regulation Theory), Reflective- Schon’s Reflection in Action/Reflection on Action. Each theoretical category was given a whole week for learning. A maximum of two learning resources per week were given as an essential read. Cognitive load theory was taught in the third course EBTL, before giving the task of lesson planning to participants. Instrumental theories were reinforced again in the last course where selection of appropriate assessment tools were discussed.

The program consisted of a physical face to face session of 5-day duration with 1 day allotted to each course, after an initial overview of course curriculum. This was conducted for all participants through interconnected workshops conducted by second and third authors in various outreach locations. The first cohort consisted of 129 students, which have increased exponentially, as the university is now running its seventh cohort; over 1700 participants from own and other institutions have so far been trained in six cohorts. Participants were recruited from different specialties to work together as a team, to encourage all three forms of participation within their community of practice (9). On Moodle, they were given tasks requiring interactions at workplace; the type of participation called “engagement.” During the face-to-face session, they met and worked with senior and junior members of other specialties, broadening their professional network; type of participation known as “alignment.” The third type of participation called “imagination” was practiced by making them reflect on their professional journey and providing individual feedback on their tasks on Moodle. This helped them in the development of their unique, personal identity (9, 10).

**Figure 1:**
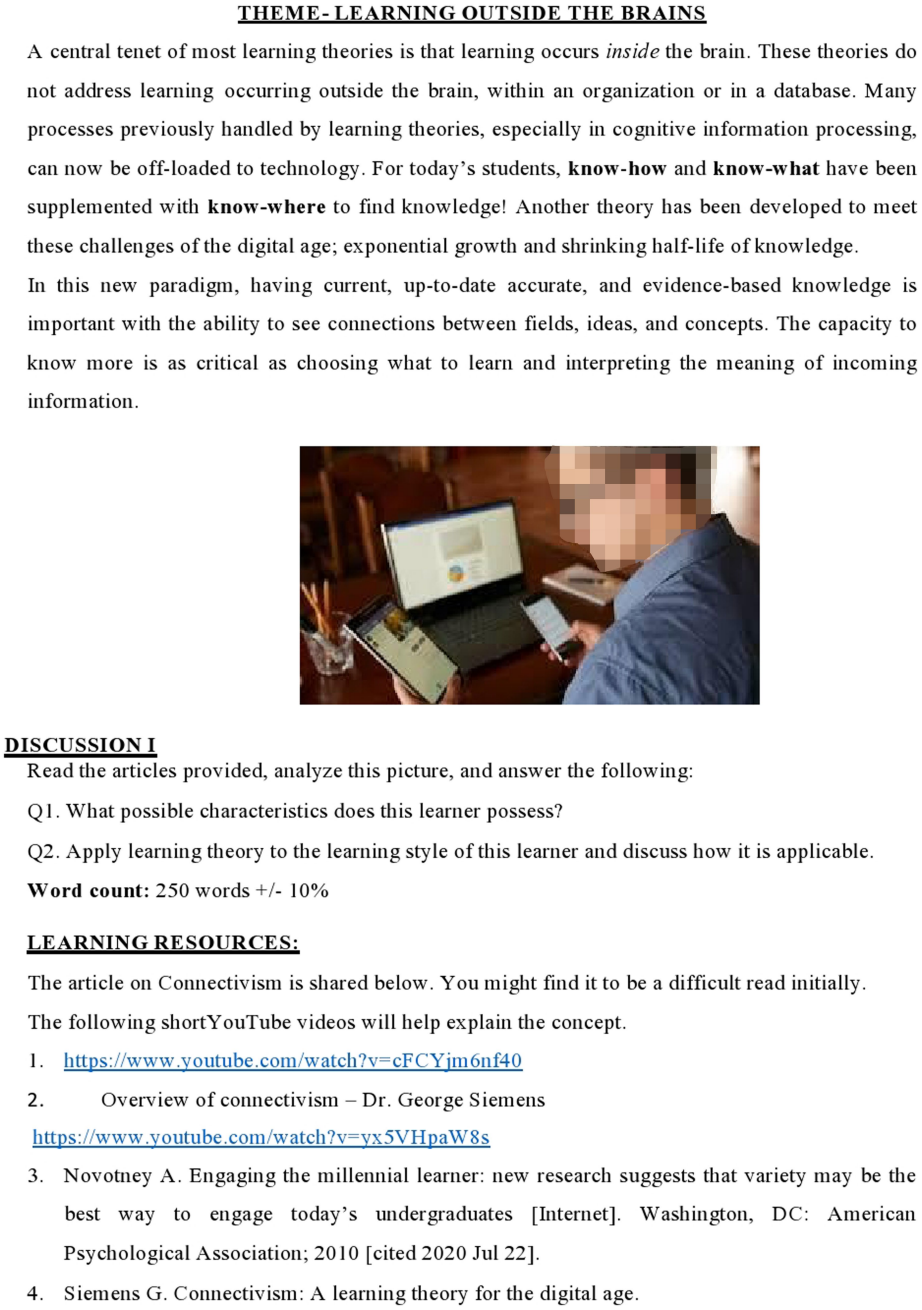
Connectivism and millennial learners – task given in week 1 of ALTA.

For the online component, the participants were divided into 11–14 distance learning groups, with one MHPE qualified educationist assigned to be the group tutor in-charge of 12 to 15 students. To ensure standardization of scoring among all the Distance Learning Study Groups (DLSGs), a course manual was developed for each of the four courses, separately. These manuals were shared with all tutors via email. A preliminary zoom meeting for orientation of participants and faculty to the course was also conducted. The educational strategies, designed and implemented within this community of practice of participants, utilized multiple learning theories (19) such as constructivism, where new knowledge was built on previous knowledge, connectivism where weekly discussions among participants were stimulated and encouraged on Moodle, behaviorism where time bound quizzes and assignments were given as tasks, and feedback was practiced. During face-to-face session, informal learning tools of experiential learning by peer interactions on group tasks, periodic reflections and mentoring were paired with community networking and interprofessional learning, to foster role modeling (13). As the goal of program was to bring transformation in identities of teachers, Mezirow’s transformational learning theory comes into play where Connectivism as a learning theory for the digital age (21), adjoined with concept of Millennial learners (22), was given as a disorienting dilemma in the first week of course ALTA (see Figure 1). This was done to challenge their old, held beliefs and practices of traditional learning in classrooms (18).

### Setting and duration of study

**Figure 2:**
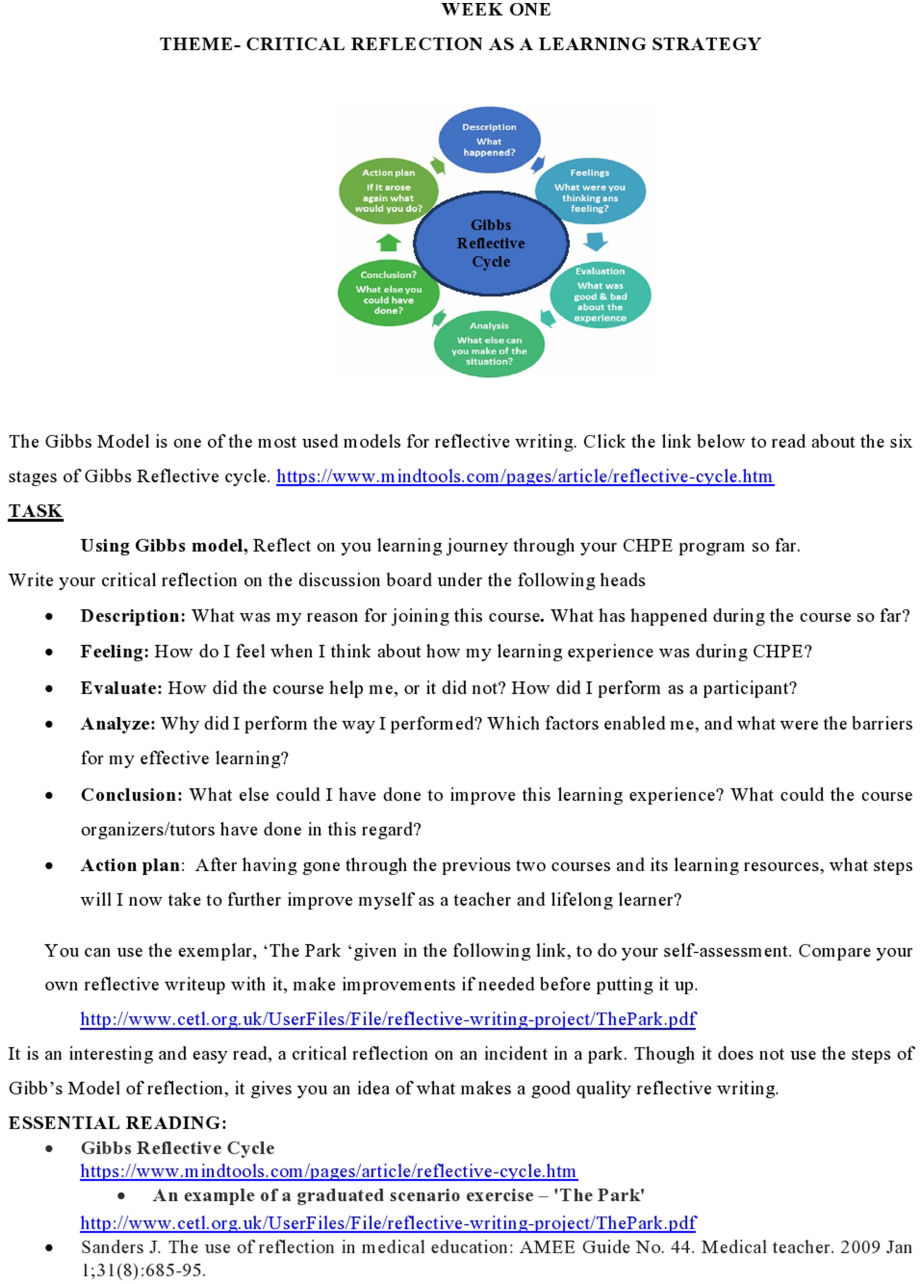
Reflective essay – task given in week 1 of EBTL.

Approval for the study was taken from IRB and Ethical committee (06/IRB & EC/NUMS/35/10397) of National University of Medical Sciences. The focus of this study is the sixth iteration of this program conducted in Fall of 2022. In the third course Evidence based Teaching and Learning (EBTL), participants were given a task of guided reflective writing (see Figure 2), on weekly interactive discussion board, from 16 Jan to 23 Jan 2023, using Gibbs reflective cycle (23). The purpose was to stimulate interactive discussion among group participants so they can reflect on their learning journey so far, and give their personal insights into whether, and how this program helped them transform their professional identities as medical teachers. By this time, all participants had attended the face-to-face session and two courses Adult Learning Theories and Application (ALTA) and Curriculum Planning and Evaluation (CPE) had been attempted.

### Sampling and study design

The target participants were medical teachers, belonging to clinical as well as basic health sciences, who were interested in developing formal skills in health professions education. All participants of certificate program in Fall 2022 cohort, who took part in weekly discussion were included in this study. Those participants who did not participate in the weekly discussion were excluded.

The study paradigm was constructionism (24). It is a philosophical paradigm which states that knowledge or meaning is built by participants in a social process, for example, discussion in a meeting, experiences at workplace. Constructionists are interested in knowing about how people interpret their experiences, how they construct their worlds, and what meaning they attribute to these experiences (24).

*Data analysis:* The study was designed to employ thematic analysis (25, 26), a flexible method for identifying, analyzing, and interpreting patterns of meanings and/or themes within a large qualitative data set. Our analysis is theory driven which is a well-structured approach, where key features of a large data set are highlighted, similarities and differences in opinions of participants are noted, collaborative insights are generated among researchers, findings are summarized and a clear, organized final report is produced (25, 26).

### Credibility

#### Thick description

Trustworthiness is achieved by analyzing data in six phases (24). In the first phase, the FK and AZ read each discussion group reflective writings in depth to capture the sense and get familiarized with the data. Subsequently, the documents were re-read (word-by-word). In the second phase, meaningful text within each reflective essay was highlighted and coded. A good code is one that captures qualitative richness of phenomenon. Subsequently, all relevant codes showing transformation in their cognition, skills, or a change they see in self or peers after doing CHPE were extracted, and their experiences were arranged in chronological order. In the third phase, the first author re-read the extracted data to make sense of the new set of extracted data relevant to the research objectives, reviewed and re-organized experiences in a correct chronological order. Iterative re-reading (back and forth re-reading process) was performed to search for themes. In the fourth and fifth phase, codes were identified into subthemes and themes. The researchers finally brought their analysis which was done without prior discussion with one another, they cross checked data codes and themes, discussed placement of excerpts within subthemes, reached consensus after discussion, and finalized the names of themes. This researchers’ triangulation lends greater credibility to the observations (27). In the sixth phase, the process of *member checking,* peer debriefing on final report and audit trailing were carried out.

#### Audit trail

To minimize inaccuracies and to visualize the processing of raw data into final themes, process logs were maintained to form an audit trail. Records of raw data, and data analysis were kept by fourth author AZ (23, 24).

#### Reflexivity

Reflexivity was managed by taking following steps: instead of taking interviews or focus groups, guided reflective writings on Moodle were taken so that participants can contemplate and reflect on their learning journey with ample time of 1 week given for the task. Though the authors have designed the reflective writing task and decided its placement during the course, there was no direct interaction between first three authors and participants during reflective writing task and its scoring as none of them were tutoring them on Moodle. The assigned group tutors, who belonged to different institutions, graded their reflective writings. This gave participants freedom to talk about their experiences in detail. Participants’ anonymity was maintained by giving them identifiers before reading their essays and quoting their excerpts.

The FK and AZ author wanted to analyze the data by looking through the lens of transformative learning theory in accordance with the goal of our program. However, we finally selected CoP as our theoretical framework, after negotiations with RA and AR. They were of the view that Mezirow’s approach lacks social, emotional, and contextual dimensions of change. The emotions, day-to-day interactions within the Community of Practice, society, the unique context of workplace, personal negotiations with all these factors, all play their role in transformation. But transformational theory only talks about beliefs challenged by disorienting dilemma and intellectual discourse causing transformation. Whereas, transformation is a complex process, and it cannot occur without social interactions, a fact agreed by Mezirow in later articles (28, 29). The cognitive processes causing transformation are still not completely understood and Transformational theory is still underconstruction (28, 29). We had planned ways to include all three types of participation within CoP of medical teachers during this course, developed interactive tasks to endorse experiential learning with peers, and fostered mentoring and feedback throughout the program, therefore, the Lave and Wenger’s theory of Community of Practice derived from situated learning seemed more appropriate to identify the processes leading to transformation (10).

## Results

Of the total 209 participants, 202 responded to discussion task.

### Identifiers for research participants

The identifiers used for the quotes of participants are F and M for gender, G for the online group assigned. Thus, F1G1 stands for first female participant of Group 1.

**Figure 3:**
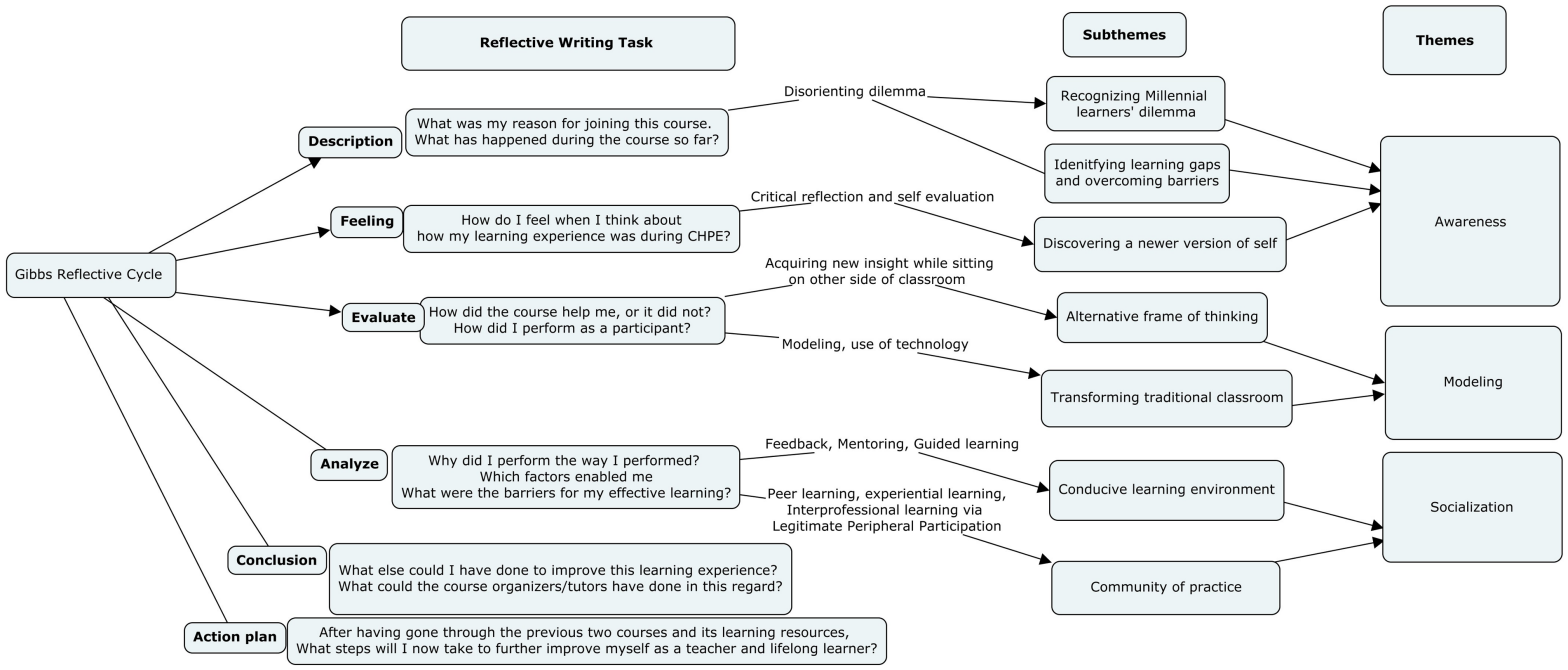
Thematic analysis of reflective essays of CHPE participants.

Based on thematic analysis of reflective essays, seven sub themes were identified showing their transformative journey which were grouped into three major themes (see Figure 3).

As a result of the six phases of analysis, ninety-one codes were produced. Next, codes that shared similar or related characteristics were headed under seven subthemes which were then grouped under three themes (see Figure 3). Finally, the subthemes and themes were linked and placed in chronological order, and the process formed a general model of transformative journey of our participants.

## Awareness

### Recognizing millennial learners’ dilemma

Millennial learners are those who were enrolled in universities at the start of the current millennium. They are born after 1982, and referred to as generation Y. Their personality, thought processes, tendencies and educational preferences are unlike their predecessors. They do not accept traditional classroom practices and educational environments (22). They are digital natives; a generation of learners who are comfortable using computers and other electronic gadgets technology because they have grown up using technology (30, 31). Therefore, engaging them in classrooms is quiet challenging for educators (22).

Introducing participants to millennial learners’ preferences to engage them was one concept thoughtfully paired with connectivism in the first week of course ALTA (see Figure 1). “*It helped me in understanding the generation z concept, liking* var*iety in the class like videos, student team-based quizzes (M5G2).”* Most participants were technologically challenged as they were digital refugees (29). Digital refugees are those whose jobs, livelihoods, and lives have been disrupted by the rapid advancement of information technology. They are not comfortable using technological devices and prefer old tools of learning (30, 31). It was challenging to make them understand the need of time “*I used to think that I and fellow students can learn* via *traditional method yet doing well why cannot this new generation. This course made me realize that new generation requires new skills and tools (M5G1).”* Learning about evolution of learning theories bridged the gap between generations, *“Now it is easy for me to understand what we were taught and how the new generation is being taught (M2G4).”* They understood the dilemma the current generation is facing, *“We are teaching 21st century students with 20th century teachers with 19th century curriculum (G14M1)”!*

### Identifying learning gaps and overcoming barriers

Participants while reflecting on their learning journey identified areas where they need hard grilling to perform their role as a teacher effectively, “*I acknowledged my ignorance about curriculum preparation. I wasn’t even looking at the forest; I was simply counting the trees (F1G3).”* They tacitly recognized the need to change their traditional teaching practices and upgrade themselves, “*This course has made me realize that our structure of medical education is quite outdated and there is strong need of updating this structure to produce competent medical professionals (F1G14).”* They realized the reason why they were adamant about their beliefs, “*The barriers to my understanding of medical education were the traditional learning techniques embedded during my undergraduate years (M4 G3).”* They acknowledged the efficiency of thoughtfully prepared interactive tasks on Moodle and during face-to-face interactions, “*The learning theories and teaching methodology taught to us are more technical compared to what I used to practice (M2G2).”*

### Discovering a newer version of self

*Participants can see a newer version of their own self “Up till now the course has given the boost, a new energy and incentive to renovate, recreate and repair myself as a facilitator of medical education (F5G4).” Through critical discourse stimuli given every week on interactive discussion forum on Moodle, they re-evaluated their teaching styles, “The course has been a catalyst to reevaluate my teaching and learning techniques (M5G8).” This cannot be achieved without reflecting upon the old practices, “I am reflecting on my knowledge and my ways of teaching, going back again and again to revise to have a critical view. In the process I have realized I have certain shortcomings. I am trying to address them (F7G4).”*

*They have acquired new skill sets, “I made a 4 weeks module of radiology which was one of the important requirements for PMDC (Pakistan Medical and Dental council-accrediting body) visit (F6G7),” designed study guides “I have developed a study guide for our coming BDS first batch all by myself (F2G5),” and took part in curriculum and assessment in their institutes, “The course so far has helped me in making MCQs, SEQs and module management (M1G13).” Their academic writing skills have improved “My vocabulary and writing have improved a lot (F3G3).” Apparently, they have become digital immigrants* (29) *“Now l have obviously more understanding of using computer while learning (F5G2).” Digital immigrants are those adults who do not like technology but learn it to meet challenges of today’s world* (30, 31).

## Modeling

### Alternative frame of thinking

Being students of CHPE, they enjoyed the attention of facilitators to their personal and professional development. The active learning strategies, use of electronic devices in classroom, continuous feedback, and reflections on their values and practices, gave them a new lens, *“Sitting on the other side of the chair re-shaped my perspective on the teaching methodologies being used (M4G13).”* They have become unconventional and gained new insight, *“I have learnt to think out of the box, create and provide the healthiest educational environment for my students and trainees (M2G8).”* They are using more advanced ways of teaching, “*It is changing my teaching style, aims and motives from traditional methods to more advanced ways. Student interaction has increased leading to better outcome of education (M3G2).”*

### Transforming traditional classroom

Kohen et al. states in his review that only 5% of faculty development programs focus on the domain of role modeling and creating awareness about this powerful teaching strategy. Most programs do not pay close attention to individuals’ professional behaviors and provision of healthy environment to the learners (13), “*As all teaching theories emphasized the importance of Role Modeling by the mentors, I am obliged to remind myself every day that a large part of curriculum is being transferred to the learners informally by the behaviors and interactions of teachers (M2G5).”*

*They took aspiration from their facilitators and willing to apply same techniques with their students “I must engage and encourage my students in their learning as I was engaged by my teachers in the course (M3G3),” “The interactive way they conducted showed me an insight and inspired me to conduct my own teaching sessions in such a manner (F5G1).” Now they try to be more friendly “Teachers are facilitators in a classroom for students, they are not dictators (M5G1).” They engage students in classrooms using technology and understand the value of e-learning, “when I go to classroom I try to connect to students and play the role of facilitator. While planning a lesson I try to focus on my objectives rather than a mere topic. I realized the importance of group discussions, integration of updated technology. How can we ask random quizzes while using Socrates in class and keeping students attentive have been a very enlightening part for me (M5G1).”*

## Socialization

### Conducive learning environment

*Participants appreciated the continuous guidance and mentoring “My course supervisor has discussed the shortcomings with me personally and has guided me well to overcome those deficiencies (F2G4)” prompt feedback, “Tutors very helpful and cooperative with timely response and grading (F1G8)” and the use of reflective practices by facilitators “I would suggest CPSP (College of Physicians and Surgeons Pakistan) to include at least one session on reflection for PG trainees (M3G4).” They appreciated the conducive learning environment provided during face-to-face session as well as on Moodle, “There is an environment of self-learning and self-development which is a useful experience (F5G4).”*

### Community of practice

*Most teachers were enrolled in the course by observing transition in their peer identity who have completed this course in previous cohort,* *“My colleagues and friends opted for CHPE, and I specifically noticed a positive change in their ideas, discussions, documentation, and use of terminologies which created a sense of curiosity in me and propelled me to take this course (F6G10).” “I used to hear terminologies from my senior colleagues who had done this course, but I only had a vague idea of what they were talking about. I thought it would be a good idea if I could also speak, understand the same language (F4G5).”*

*They enjoyed interprofessional learning during face-to-face session, “Meeting with professionals of different specialties at the forum of CHPE from different backgrounds was an excellent experience (M3G4).” The helpful teachers and seniors made their journey easier, “The most adaptable course design, my peers, my seniors, and the learning resources all contributed to my motivation, insight, and success in the course (M1G2).”*

## Discussion

The three themes manifest three major processes essential for identity transformation: Awareness, Modeling, and Socialization within Community of Practice (9, 18). These processes, when present together help an individual sail through unchartered waters smoothly, giving a new perspective of thinking to appreciate the hidden side of the voyage. This new perspective which is central to this anticipated phenomenon of transformation is gained through critical reflection (18).

The change occurs where pre-held assumptions or beliefs are questioned or do not work in the face of an event or crisis. In response to this event or crisis, emerges an unease or sense that things do not fit and that a change is needed, existing meanings need to be redefined, or new frame of reference is to be established (32). This new frame of reference or perspective transformation has three dimensions: psychological (changes in understanding of the self), convictional (revision of belief systems), and behavioral (changes in lifestyle) (33). In contrast, the CoP model proposed by Lave and Wenger (9) addresses transformation of identity because of interaction, information sharing by engaging with members of community in an informal context, and identifying themselves as part of the group, co-responsible for the collective learning through practice, described as peripheral participation, a key process in learning (9).

A disorienting dilemma is a challenge and/or a crisis that shakes a person’s old held beliefs on a certain area, triggers a questioning of assumptions, and stimulates reflections resulting in transformed beliefs after deliberation (33). It is also interpreted as “setting the stage” for readiness to change (32). At first, learning theories in context of millennial learners’ personality and preferences were presented as a disorienting dilemma (32) to challenge the participants who, then entered critical discourse with other members. Modeling of interactive learning and digitalization of classrooms by facilitators made them reflect onto their traditional ways. This raised an inner need in them to upgrade their teaching methodologies. It was, indeed, a rate limiting step of the whole transformative process. In our CHPE program, nearly all participants were digital refugees (31), treading on traditional grounds familiar to them since their student life. By enrolling in this program, they entered a “foreign territory” where they were challenged by unfamiliar format of a hybrid program and strange perspectives of learning in learning theories. This alienation pushed them towards the second order reflection essential for transformation (34). Intellectual discourses, on Moodle and during contact session, with members of Community of Practice (CoP) transformed our participants from peripheral legitimate participation to become autonomous thinkers, who will become change agents in their respective organizations. This rich, multi-dimensional process of interprofessional learning and professional socialization enhanced the learning impact (9). They were guided by the facilitators who were not mere academic teachers but one step ahead, i.e., mentors who take part in their informal, unconscious learning by modeling (7) so that gradually they will acquire the same way of talking, questioning and an analogous way of understanding. All this process of learning is similarly described in literature (18).

Results of our study reinforced the findings of a recent study in Pakistan who talked about the positive impact of conscientious and explicit role modeling by the medical faculty in teaching professionalism (35). It highlighted the greater influence of positive role modeling within community of practice on professionalism of medical students and placed emphasis on need for more faculty development programs which can facilitate and support medical educators as role models (35). The format of our program followed principles mentioned in a recent study of Indonesia, which are effective for professional identity transformation of medical teachers. They talked about the use of early socialization, experiential learning, and envisioning the future by reflections to make a faculty development program motivating and inspiring (36). Our study helped researchers to understand the stance of Kember and Kwan (37) and de Jonge et al. (38) who explained that faculty development falls short of changing pedagogical practices when educational beliefs of educators are not transformed, and a conducive learning environment is not created. Our study results explicitly reinforced this paradigm that educational reforms can only be brought-in by faculty development interventions specifically designed to change educational beliefs of teachers about teaching and learning practices.

### Recommendations

Faculty development programs must be intense, longitudinal, and carry an individual approach of focusing on the professional identity development of medical teachers, by working on their beliefs, values, and practices. This can be achieved by incorporating informal teaching practices of modeling, mentorship, experiential learning within community of practice (13, 39), and by fostering a conducive learning environment where self-evaluation and self-regulation is promoted. The process is augmented by formal teaching tools of reflective exercises, online learning, peer coaching and feedback (38, 39). Evaluation of faculty development programs need qualitative data collection tools to understand the process of change occurring in participants (13, 39).

## Conclusion

Our CHPE program has transformed a few aspects of the professional identity of our participants. The journey was rewarding as they underwent critical discourse and reflections when challenged by millennial learners” dilemma. They developed new insight into their teaching practices and discovered a newer version of self by acquiring alternative frame of thinking. Technophobic participants became digital immigrants and traditional classrooms were transformed to unconventional ones. They started using digital learning aids, interactive learning strategies, and incorporated formative assessment, e-quizzes, and feedback in their teaching practices. They appreciated role modeling by facilitators, enjoyed interprofessional learning within Community of Practice of educators, flexible timings, providence of weekly learning resources and following deadlines of given tasks. Consequently, they underwent self-evaluation, practiced self-regulation, and developed critical thinking skills and self-awareness. They started developing mini courses and assessments in their institutes. They also moved from external motivation of job stay and certificate achievement, which was needed for their promotion, towards internal motivation of being a successful mentor and an influencer for the students.

### Limitations

The program is uniquely designed after needs assessment in a national context. As the first author has primarily designed the course ALTA, second and third authors designed the whole CHPE program, their biases may have influenced the interpretations of results. Our study has evaluated only the individual impact of program on participants and has not evaluated the organizational impact. As the reflective essays are taken only from participants of sixth cohort of the program, the applicability of our findings to previous cohorts cannot be predicted. Moreover, the socio-cultural and demographic characteristics of our education system are unique and may not be applicable to other educational systems.

## Data availability statement

The original contributions presented in the study are included in the article/supplementary material, further inquiries can be directed to the corresponding author.

## Ethics statement

The studies involving humans were approved by IRB and Ethical committee (06/IRB & EC/NUMS/35/10397) of National University of Medical Sciences. The studies were conducted in accordance with the local legislation and institutional requirements. The Ethics Committee/Institutional Review Board waived the requirement of written informed consent for participation from the participants or the participants’ legal guardians/next of kin because it was a reflective writing given as a weekly discussion task in a course on Moodle.

## Author contributions

FK: Conceptualization, Formal analysis, Investigation, Methodology, Supervision, Writing – original draft, Writing – review & editing. RA: Writing – original draft. AR: Formal analysis, Methodology, Supervision, Writing – review & editing. AZ: Data curation, Formal analysis, Investigation, Methodology, Project administration, Writing – review & editing.
